# Lifetime violence and suicidal ideation among young women (18–24 years) in Uganda: Results from a population-based survey

**DOI:** 10.3389/fgwh.2023.1063846

**Published:** 2023-04-17

**Authors:** Peter Kisaakye, Agatha Kafuko, Paul Bukuluki

**Affiliations:** ^1^ Department of Population Studies, School of Statistics and Planning, Makerere University, Kampala, Uganda; ^2^ Department of Social Work and Social Administration, School of Social Sciences, Makerere University, Kampala, Uganda

**Keywords:** lifetime violence, suicidal ideation, young women, Uganda, population-based survey

## Abstract

**Introduction:**

Violence is a major global public health issue that threatens the physical and mental health of victims. Of particular concern is the increasing evidence which suggests that violence is strongly associated with suicidal behavior including ideation.

**Methods:**

This study uses data from the 2015 Violence Against Children Survey (VACS). This study seeks to highlight the relationship between lifetime violence and suicidal ideation using a nationally representative sample of 1,795 young women (18–24 years) in Uganda.

**Results:**

Results indicate that respondents who experienced lifetime sexual violence (aOR = 1.726; 95%CI = 1.304–2.287), physical violence (aOR = 1.930; 95%CI = 1.293–2.882) or emotional violence (aOR = 2.623; 95%CI = 1.988–3.459) were more likely to experience suicidal ideation. Respondents who were not married (aOR = 1.607; 95%CI = 1.040–2.484), not having too much trust with community members (aOR = 1.542; 95%CI = 1.024–2.320) or not having a close relationship with biological parents (aOR = 1.614; 95%CI = 1.230–2.119) were more likely to experience suicidal ideation. Respondents who did not engage in work in the past 12 months prior to the survey (aOR = 0.629; 95%CI = 0.433–0.913) were less likely to experience suicidal ideation.

**Conclusion:**

The results can be used to inform policy and programming and for integration of mental health and psychosocial support in programming for prevention and response to violence against young women.

## Background

Violence is a major global public health issue that threatens the physical and mental health of victims (1). Every year, 1 billion children are affected by a range of forms of violence including physical, sexual and emotional violence (2). Childhood violence is associated with lifelong impact on the individual including increased risk of injury, infectious diseases, mental health problems, reproductive health problems, and non-communicable diseases, as well as damage to the body's nervous, endocrine, circulatory, musculoskeletal, reproductive, respiratory, and immune systems (3).

Studies have established that childhood violence potentially has adverse effects on the mental wellbeing of victims (4, 5). Of particular concern is the increasing evidence which suggests that violence is strongly associated with suicidal behavior including ideation (6, 7). Suicidal behavior is a global public health problem that affects 700,000 people annually (8). In 2019, 1.3% of all deaths were by suicide and 77% of these were in low-and-middle income countries (LMIC) (8). Suicide is the leading cause of death among the population aged 15 to 24 years old globally (9). In Uganda studies on suicide among young people are based on community, clinical and school samples. The prevalence of suicidal ideation was 6.3% in a sample of university students (10); another study found that the prevalence of suicidal ideation among adolescents was as high as 30.6% (11). Among the refugees hosted in Uganda, the prevalence of suicidal ideation is 5% (12). A range of factors are associated with suicidal ideation including financial difficulties (13), family breakdown or conflicts (14), and trauma (11), unemployment (10).

A recent systematic review and meta-analysis found that sexual abuse, physical abuse, emotional abuse, emotional neglect, physical neglect, and combined abuse were significantly associated with higher rates of suicide attempts (7). Moreover, young women in Uganda have been observed to experience violence more than their male counterparts (15). Yet, there is limited research on the effect of violence on suicidal ideation among vulnerable young women in developing countries (16). This study therefore seeks to highlight the relationship between lifetime violence and suicidal ideation among young women (18–24 years) in Uganda, using a nationally representative dataset. While the main focus is to highlight the relationship between lifetime violence and suicidal ideation, the analysis also controlled for other key variables to see how the effect changes. We focus on young women (18–24 years) because it is the age group that responded to questions on lifetime violence (ever experienced any form of violence). We focus on suicidal ideation because it is more common than attempted or completed suicide (17). Moreover, suicidal ideation is often a precursor to attempted or completed suicide (18–20). The results from such analyses can help design strategic and integrated programs that address the risks that occur simultaneously, related to violence and suicidal ideation among vulnerable women.

## Data and methods

### Source of data

This study uses data from the 2015 Violence Against Children Survey (VACS). We use the 2015 survey data because it is the latest publicly available nationally representative dataset. The VACS is a nationally representative cross-sectional population-based household survey that targeted adolescents and young people in the age group 13–24. Information from respondents that were aged 18–24 years was used to compute lifetime violence. For the purpose of this study, the analyses are restricted to only female respondents that were aged 18–24 years at the time of the survey.

### Sample size, sampling and data collection

The sample included in the analyses is 1,795 female respondents aged 18–24 years. A three-stage cluster sampling design was employed. The first stage involved selecting primary sampling units which were used as a sampling frame. The second stage of sampling involved listing all households in the selected primary sampling unit. Twenty-five households were selected from each sampling unit using probability systematic sampling method. The third stage involved randomly selecting an individual for interview from the selected household. A split sampling approach was used to select female and male respondents. This was intended to protect the confidentiality of respondents and eliminate the chance of the perpetrator and the one sexually assaulted being both interviewed. Data were collected using a face-to-face structured questionnaire programmed on a tablet by well-trained interviewers. Interviewers were trained on the research protocol procedures and ethics in research. During data collection, same-sex interviews were conducted: where female interviewers interviewed female respondents and male interviewers interviewed male respondents (21).

### Research ethics

The Makerere University College of Health Sciences ethics review committee and The Uganda National Council for Science and Technology and the Centre for Disease Control (CDC) Institutional Review Board independently reviewed and provided approval to conduct the study. All respondents provided informed consent to participate in the study. However, the data used in this study is publicly available and approval was not required for its re-use.

## Measurement of variables

### Dependent variable

Measurement of suicidal ideation was measured using the question: “Have you ever thought about killing yourself?”. Response options for the question were “yes” or “no”.

### Independent variables

Table 1 summarizes the independent variables and measures included in this study.

**Table 1:** Independent variables and measures.

Variables	Measures
Sexual violence	Experience of unwanted sexual touching (fondling, pinching, grabbing, or touching on or around your sexual body parts, attempted unwanted sexual intercourse (perpetrator attempted intercourse through harassment, threats, and trick but penetration did not occur), pressured intercourse (unwanted sex was completed through use of threats or non-physical pressure), or physically forced sex (unwanted intercourse completed through physical force). Respondents who experienced any of these events were categorized as having experienced lifetime sexual violence, otherwise no.
Physical violence	Experience of being punched, kicked, whipped, or beaten with an object, choked, suffocated, tried to drown, or burned intentionally or used or threatened with knife, gun or other weapon on them. Respondents who experienced any of these events were categorized as having experienced lifetime physical violence, otherwise no.
Emotional violence	Experience of being told that you were not loved, or did not deserve to be loved, being told that they wished you had never been born or were dead or ridiculed or put down. Respondents who experienced any of these events were categorized as having experienced lifetime emotional violence, otherwise no.
Highest level of education	Less than primary, primary, secondary, higher than secondary, not stated
Religion	Catholic, Protestant, Muslim, Pentecostal, Seventh Day Adventist, other
Orphan status	Not orphaned, one parent dead, both parents dead
Engaged in work in the last 12 months	Yes, no, not stated
How much the respondent talked to their friends	A lot, a little, not very much, not at all, not stated
Current marital status	Currently married, not currently married, not stated
Justified gender-based physical violence (whether it is right for a man to hit his wife)	If she goes out without telling him, if she does not take care of the children, if she argues with him, if she refuses to have sex with him, if she burns the food. A response to each of this is yes, no, or not stated. Respondents who answered yes to any of the statements were categorized as having justified physical violence, otherwise no.
Trust people living in your community	A lot, some, not too much, not at all, not stated
Nature of the relationship with biological parents	Close relationship, no relationship, not stated

### Data analysis

Data analysis was performed using the Stata software version 15 (22). Three models were run. The first model (Model 1) only controlled for violence (sexual, physical, and emotional) on suicidal ideation. The second model (Model 2) generated unadjusted odds ratios for all variables included in the study. Model 3 generated adjusted odds ratios for all variables included in the study.

## Results

### Characteristics of respondents

Table 2 shows the characteristics of young women (18–24 years). About four out of every ten respondents (45.6%) had primary level of education. Most respondents (37.1%) were Catholic. Slightly more than half of the respondents (52.8%) had engaged in work in the past 12 months prior to the survey or were currently married at the time of the survey (53.5%). Table 2 shows that 56.5% of respondents justified violence (husbands physically abusing their partners). About sixty-five percent of respondents reported to have a close relationship with their biological parents.

**Table 2:** Distribution of respondents.

Variable	Frequency	Percent	95% CI
Highest level of education			
Less than primary	84	4.7	3.2–6.8
Primary	818	45.6	40.0–51.2
Secondary	463	25.8	21.7–30.3
Higher than secondary	65	3.6	2.2–5.9
Not stated	364	20.3	16.9–24.2
Religion			
Catholic	667	37.1	31.5–43.2
Protestant	568	31.6	27.0–36.7
Muslim	249	13.9	10.0–19.0
Pentecostal	257	14.3	10.5–19.2
Seventh Day Adventist	52	2.9	0.9–8.7
Other	3	0.2	0.0–0.8
Orphan status			
Not orphan	681	38.0	34.0–42.1
One parent died	70	3.9	2.6–5.8
Both parents died	2	0.1	0.0–0.4
Not stated	1042	58.1	53.6–62.4
Engaged in work (past 12 months)			
Yes	948	52.8	47.6–58.0
No	425	23.7	18.7–29.5
Not stated	422	23.5	19.3–28.3
Talk to friends			
A lot	359	20.0	16.7–23.8
A little	637	35.5	31.3–39.9
Not very much	467	26.0	21.9–30.6
Not at all	328	18.3	14.6–22.6
Not stated	3	0.2	0.0–1.0
Current marital status			
Currently married	960	53.5	48.0–58.9
Not currently married	153	8.5	6.4–11.2
Not stated	682	38.0	32.2–44.1
Justification of physical violence			
Yes	1,014	56.5	51.1–61.7
No	747	41.6	36.7–46.7
Not stated	34	1.9	1.0–3.6
Trust community members			
A lot	278	15.5	11.9–19.9
Some	464	25.8	21.9–30.2
Not too much	652	36.3	32.7–40.1
Not at all	393	21.9	18.2–26.0
Not stated	9	0.5	0.2–1.6
Relationship to biological parents			
Close relationship	1,171	65.2	61.0–69.2
No close relationship	509	28.4	24.8–32.2
Not stated	115	6.4	4.7–8.8
Total	1,795	100	

CI, Confidence Interval.

### Experience of lifetime violence among young women (18-24 years)

**Figure 1:**
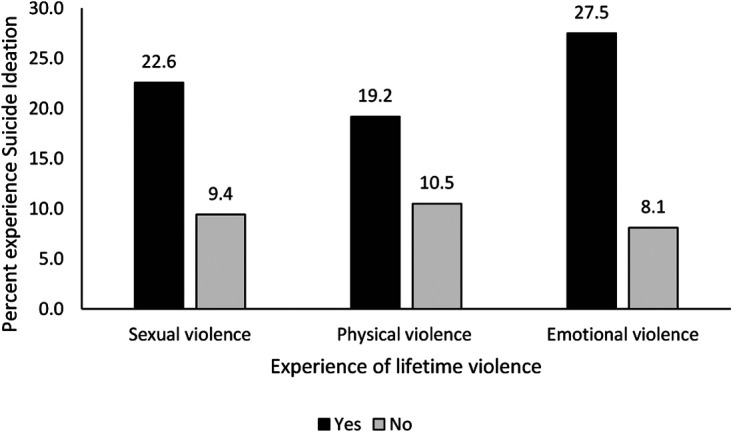
Experience of lifetime violence and suicidal ideation.

Overall, 72.1% of respondents reported to have experienced physical violence. Slightly more than half (52.0%) of respondents experienced sexual violence and 42.6% reported to have experienced emotional violence. Figure 1 shows that experience of violence (sexual, physical, or emotional) was associated with experience of suicidal ideation. Respondents who experienced sexual violence (22.63% vs. 9.4%), physical violence (19.2% vs. 10.5%) or emotional violence (27.5% vs. 8.1%) were more likely to report higher rates of lifetime suicidal ideation than respondents who did not experience such violence The results in Figure 1 show that suicidal ideation was observed more among respondents who experienced emotional violence (27.5%) compared to other forms of violence: sexual (22.6%) or physical (19.2%).

### Factors associated with suicidal ideation

The results in Table 3 show results from a model (Model 1) that controlled for only sexual, physical, and emotional violence. The results indicate that sexual (OR = 1.959; 95%CI = 1.492–2.575), physical (OR = 1.842; 95%CI = 1.238–2.741), and emotional violence (OR = 3.054; 95%CI = 2.328–4.007) were all significantly associated with suicidal ideation. This means that respondents who experienced lifetime sexual, physical, or emotional violence were more likely to experience suicidal ideation than respondents who did not experience any lifetime violence. However, the results indicate that the risk of experiencing suicidal ideation is higher among respondents who experienced emotional violence than other forms of violence (sexual and physical).

**Table 3:** The relationship between lifetime violence and suicidal ideation (model 1).

Variable	Unadjusted odds (95%CI)
Sexual violence (RC = No)	
Yes	1.959 (1.492–2.575)***
Physical violence (RC = No)	
Yes	1.842 (1.238–2.741)**
Emotional violence (RC = No)	
Yes	3.054 (2.328–4.007)***

** = *p* < 0.01.

*** = *p* < 0.001.

RC, Reference categories.

Table 4 shows results from Model 2 (unadjusted odds) and Model 3 (adjusted odds). The results from Model 2 and Model 3 point to the same pattern and direction. After controlling for all background variables (adjusted odds) in the model (Model 3) other than those that measure violence, the results indicate that respondents who experienced lifetime sexual violence (aOR = 1.726; 95%CI = 1.304–2.287), physical violence (aOR = 1.930; 95%CI = 1.293–2.882) or emotional violence (aOR = 2.623; 95%CI = 1.988–3.459) were more likely to experience suicidal ideation than respondents who did not experience sexual, physical or emotional violence.

**Table 4:** Factors associated with suicidal ideation.

Variable	Unadjusted odds (95%CI)	Adjusted odds (95%CI)
	Model 2	Model 3
Sexual violence (RC = No)		
Yes	1.959 (1.492–2.575)***	1.726 (1.304–2.287)***
Physical violence (RC = No)		
Yes	1.842 (1.238–2.741)**	1.930 (1.293–2.882)**
Emotional violence (RC = No)		
Yes	3.054 (2.328–4.007)***	2.623 (1.988–3.459)***
Highest level of education (RC = Less than Primary)		
Primary	1.052 (0.635–1.746)	1.257 (0.748–2.111)
Secondary	1.150 (0.676–1.959)	1.234 (0.712–2.136)
Higher than secondary	1.541 (0.747–3.177)	2.118 (0.985–4.553)
Not stated	0.703 (0.396–1.249)	0.802 (0.439–1.468)
Religion (RC = Catholic)		
Protestant	1.102 (0.832–1.462)	1.111 (0.833–1.482)
Muslim	0.769 (0.479–1.235)	0.656 (0.398–1.079)
Pentecostal	1.165 (0.808–1.679)	1.102 (0.759–1.602)
SDA	1.556 (0.684–3.540)	1.721 (0.741–3.994)
Other	2.171 (0.533–8.841)	2.073 (0.490–8.770)
Orphan status (RC = Not orphan)		
One parent died	1.481 (0.803–2.732)	1.521 (0.807–2.864)
Both parents died	-	-
Not stated	1.409 (1.073–1.851)*	1.338 (0.991–1.807)
Engaged in work (past 12 months) (RC = Yes)		
No	0.531 (0.371–0.759)**	0.629 (0.433–0.913)*
Not stated	1.168 (0.884–1.544)	1.103 (0.819–1.486)
Talk to friends (RC = A lot)		
A little	0.954 (0.677–1.346)	0.953 (0.669–1.359)
Not very much	1.293 (0.915–1.826)	1.067 (0.746–1.524)
Not at all	1.001 (0.656–1.529)	1.092 (0.701–1.701)
Not stated	1.900 (0.261–13.843)	3.380 (0.406–28.115)
Current marital status (RC = currently married)		
Not currently married	2.039 (1.355–3.068)**	1.607 (1.040–2.484)*
Not stated	1.444 (1.119–1.865)**	1.378 (1.031–1.841)*
Justification of physical violence (RC = No)		
Yes	1.296 (0.996–1.687)	1.214 (0.922–1.597)
Not stated	2.140 (0.864–5.298)	1.827 (0.713–4.682)
Trust community members (RC = A lot)		
Some	1.450 (0.940–2.237)	1.301 (0.830–2.039)
Not too much	2.272 (1.548–3.332)***	1.542 (1.024–2.320)*
Not at all	2.101 (1.358–3.251)**	1.433 (0.901–2.279)
Not stated	1.150 (0.157–8.417)	0.943 (0.123–7.258)
Relationship to biological parents (RC = Close relationship)		
No close relationship	2.199 (1.695–2.854)***	1.614 (1.230–2.119)**
Not stated	2.869 (2.001–4.115)***	2.015 (1.371–2.961)***

* = *p* < 0.05.

** = *p* < 0.01.

*** = *p* < 0.001.

The results in Table 4 show that respondents who did not engage in work in the past 12 months prior to the survey (aOR = 0.629; 95%CI = 0.433–0.913) were less likely to experience suicidal ideation than respondents who engaged in work in the past 12 months. Not being married (aOR = 1.607; 95%CI = 1.040–2.484) was associated with a more likelihood to experience suicidal ideation than being married. Respondents who reported not to have too much trust with community members (aOR = 1.542; 95%CI = 1.024–2.320) were more likely to experience suicidal ideation than respondents who reported to have a lot of trust with community members. The results from Model 3 indicate that young women (18-24 years) who reported not to have a close relationship with their biological parents (aOR = 1.614; 95%CI = 1.230–2.119) were more likely to experience suicidal ideation than respondents who reported to have a close relationship.

## Discussion

Our results demonstrate that lifetime violence among young women is high with physical and sexual violence being the most prevalent. These findings are in line with results reported elsewhere (23, 24). Our results demonstrate a significant association between the different forms of violence (physical, emotional, and sexual violence) and suicidal ideation. These results are in line with several studies that have demonstrated a significant relationship between violence and psychosocial distress (25, 26) including suicidal ideation. The reality that young women who experience lifetime sexual, physical, or emotional violence are more likely to experience suicidal ideation underscores the risk violence poses to mental health and psychosocial wellbeing of young women (25, 27). It also underscores the need to integrate mental health and psychosocial support interventions in policy and programming addressing prevention and response to violence against women (28).

The results indicate that the risk of experiencing suicidal ideation is highest among those that experienced emotional violence, followed by sexual violence and the risk is lowest among those that experienced physical violence. This finding resonates with previous findings that report a strong relationship between emotional violence and suicidal ideation (29–31). Emotional abuse can often lead to trauma (30), with lasting consequences which may lead to mental distress especially among young people (32). The results show that being engaged in work was associated with a likelihood to experience suicidal ideation. This may contradict some of the findings that associate access to employment and positive psychosocial outcomes. However, this needs to be understood in the context in which our respondents work. In some of these contexts the workplace could also be one of the sites of violence involving working under hazardous conditions and lack of protective workplace policies (33, 34). Particularly for young women, balancing work pressure and gender roles at home—that has been described as unpaid care work—could also contribute to stress more so in some work settings where childcare services are not well developed (35). Results show that the risk of experiencing suicidal ideation was higher among those who were not currently married. Evidence on the relationship between marriage and suicidal ideation is not conclusive in the literature; some studies have shown that marriage has no influence on suicidal ideation (17). However, it could be that having a companion or spouse (depending on the quality of the relationship) is more likely to build resilience and positive coping mechanisms, but this may be dependent on the context of the relationship between a couple and its quality. Social capital in the form of having a network of community members young women trust was associated with a less likelihood to experience suicidal ideation. Similar findings about the relationship between trust and suicidal ideation have been reported elsewhere (36–38). This further emphasizes the importance of social capital, particularly social networks, in facilitating better coping and mental health for young women (39, 40).

The results show that a positive quality of relationships (close relationships) between young women and their biological parents is associated with a decreased risk of developing suicidal ideations. This is similar to other studies that underscore the contribution of attachment and relationships with family members (especially parents) in facilitating mental health and psychosocial wellbeing (41).

This study has four major limitations. First, this paper uses a single measure of suicidal ideation; yet, there could be a diversity in the measurement scales of suicidal ideation. Second, we are unable to infer causality from the cross-sectional research design: we note the possibility of suicidal ideation occurring either before or after experiencing violence—which we cannot ascertain. Third, the data used in this study may not be free from recall bias; it is possible that those with a history of suicidal ideation may be more likely to recall experiences of violence in their lifetime, or, may have a heightened memory of such events, or may attribute greater salience to past instances of violence. Fourth, although we use the most recent nationally representative dataset available, we recognize the fact that the data were collected 8 years ago, and rates of violence and suicidal ideation could have changed since then.

The results reported in this study demonstrate that young people experience psychosocial problems that may be caused by violence. These results demonstrate the importance of ensuring that policy and programming for prevention and mitigation of violence should integrate strong elements of mental health and psychosocial support especially among young people. Programmes working with young people should endeavor to conduct risk and needs assessments for psychosocial problems. The results of this study further highlight the need to integrate mental health and psychosocial programming in all projects targeting young people.

## Data Availability

The original contributions presented in the study are included in the article/Supplementary Material, further inquiries can be directed to the corresponding author/s.
